# Supplementary material for: Modulation of p75NTR on Mesenchymal Stem Cells Increases Their Vascular Protection in Retinal Ischemia-Reperfusion Mouse Model
Source: Int J Mol Sci. 2021 Jan 15;22(2):829. doi: 10.3390/ijms22020829 (PMC7830385; doi:10.3390/ijms22020829)
Supplement: Supplementary file 1 [file ijms-22-00829-s001.pdf]

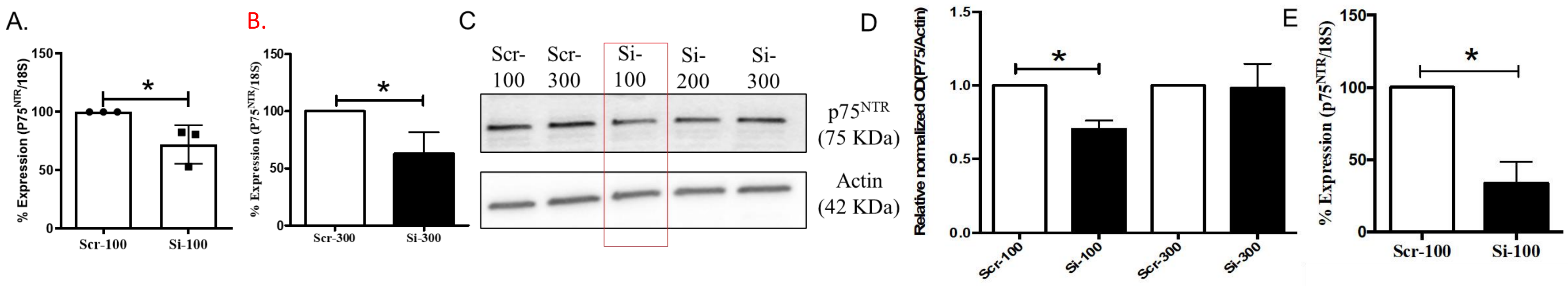

Figure S1. Transfection efficiency of $p75^{NTR}$ in MSCs. (A and **B)** Quantitative real-time PCR of *$p75^{NTR}$* mRNA expression in GFP-labeled MSCs lysates showing significant decrease by either 100 or 300 nM concentrations with no significant difference in between both concentrations (*, significant using un-paired Student t-test, *n* = 3). **(C-D)** Representative Western blotting and bar graph of $p75^{NTR}$ protein expression in GFP-labeled MSCs lysates showing significant decrease with 100nM but not 300 nM concentration (*, significant using un-paired Student T-test, *n* = 4–7).**(E)** Quantitative real-time PCR of *$p75^{NTR}$* mRNA expression in non GFP-labeled MSCs lysates showing significant decrease (*, significant using un-paired Student t-test, *n* = 3).

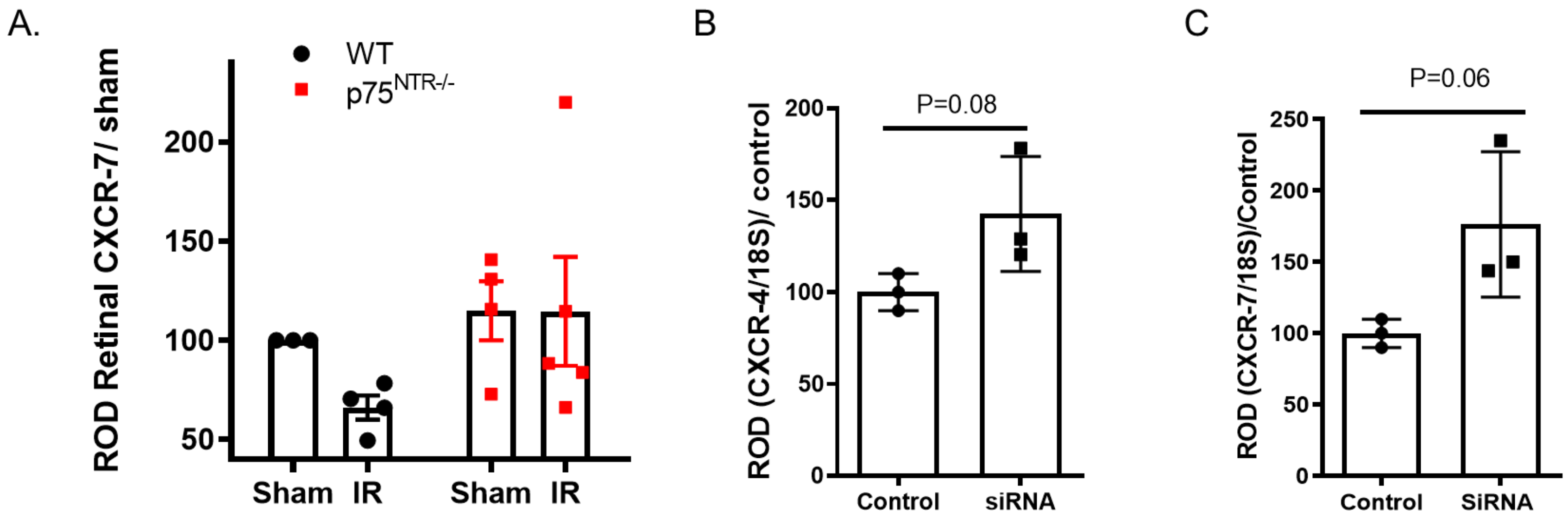

Figure S2. Genetic deletion of *p75*^{NTR} increased gene expression of SDF-1α receptors in ischemic retinas and MSCs. (A) Bar graph and statistical analysis for quantitative real-time PCR of mRNA expression of *CXCR-7* in WT and p75$^{NTR-/-}$ retinas subjected to I/R. I/R tended to decrease gene expression of CXCR-7 in WT retinas, an effect that was not observed in p75$^{NTR-/-}$ retinas (n = 3–4). **(B** and **C)** Bar graph and statistical analysis for quantitative real-time PCR in GFP-MSCs showing a trend of increased mRNA expression of *CXCR-4* and *CXCR-7* upon silencing *p75*^{NTR} expression (n = 3).
